# Proteome-Wide Analysis of Autoantibodies in Open-Angle Glaucoma in Japanese Population: A Pilot Study

**DOI:** 10.3390/biomedicines13030718

**Published:** 2025-03-14

**Authors:** Naoko Takada, Makoto Ishikawa, Kota Sato, Hiroshi Kunikata, Takahiro Ninomiya, Akiko Hanyuda, Eriko Fukuda, Kei Yamaguchi, Chihiro Ono, Tomoko Kirihara, Chie Shintani, Chihiro Tsusu, Aki Osanai, Naoki Goshima, Yukitoshi Izumi, Charles F. Zorumski, Toru Nakazawa

**Affiliations:** 1 Department of Ophthalmology, Tohoku University Graduate School of Medicine, Sendai 980-8574, Japan; naoko.takada.d7@tohoku.ac.jp (N.T.); makoto.ishikawa.c2@tohoku.ac.jp (M.I.);; 2 Opthalmic Imaging and Information Analytics, Tohoku University Graduate School of Medicine, Sendai 980-8574, Japan; 3 Department of Advanced Ophthalmic Medicine, Tohoku University Graduate School of Medicine, Sendai 980-8574, Japan; 4 Department of Retinal Disease Control, Ophthalmology, Tohoku University Graduate School of Medicine, Sendai 980-8574, Japan; 5 Department of Ophthalmology, Keio University School of Medicine, Shinanomachi, Shinjuku-ku, Tokyo 160-8582, Japan; 6 Epidemiology and Prevention Group, Center for Public Health Sciences, National Cancer Center, Tokyo 104-0045, Japan; 7 ProteoBridge Co., Tokyo 135-0064, Japan; 8 Molecular Profiling Research Center for Drug Discovery, National Institute of Advanced Industrial Science and Technology (AIST), Tokyo 135-0064, Japan; 9 Cellular and Molecular Biotechnology Research Institute, National Institute of Advanced Industrial Science and Technology (AIST), Ibaraki 305-8566, Japan; 10 Ophthalmic Innovation Center, Santen Pharmaceutical Co., Ltd., Osaka 530-0011, Japan; 11 Product Development Division, Santen Pharmaceutical Co., Ltd., Nara 630-0101, Japan; 12 Taylor Family Institute for Innovative Psychiatric Research, Washington University School of Medicine, St. Louis, MO 63110, USA; 13 Center for Brain Research in Mood Disorders, Washington University School of Medicine, St. Louis, MO 63110, USA; 14 Department of Psychiatry, Washington University School of Medicine, St. Louis, MO 63110, USA

**Keywords:** autoantibody, open-angle glaucoma, proteome-wide analysis

## Abstract

**Objectives:** The objective of this study was to identify novel autoantibodies specific for open-angle glaucoma (OAG), including normal-tension glaucoma (NTG) and primary open-angle glaucoma (POAG), using proteome-wide autoantibody screening and to determine their utility for diagnosis. **Methods:** We conducted proteome-wide autoantibody screening by wet protein arrays. Autoantibody reactivity in the plasma of OAG patients (50 NTG and 69 POAG patients) was quantitatively analyzed and compared to that of controls (35 cataract patients). The area under the curve (AUC) of the receiver operating characteristic (ROC) and multivariate analyses were used to determine diagnostic potential in patients with OAG. **Results:** Based on differences in autoantibody titers and positivity rates, four autoantibodies against ETNK1, VMAC, NEXN, and SUN1 were selected as potential biomarkers to discriminate OAG and cataract. In discrimination between POAG and cataract, the AUCs of ETNK1 and VMAC were calculated to be 0.820 (95%CI: 0.733–0.907) and 0.889 (95%CI: 0.818–0.959), respectively. Furthermore, the combination of these four antibodies demonstrated diagnostic potential for OAG with an AUC of 0.828 (95%CI: 0.757–0.898) by multivariate analysis. **Conclusions:** Four new glaucoma-associated autoantibodies were identified in this study. The differences in autoantibody patterns in the plasma between glaucoma and cataract patients support their potential utility as biomarkers for glaucoma screening.

## 1. Introduction

Glaucoma is characterized by irreversible and progressive damage to retinal ganglion cells (RGCs) [1] and is the leading cause of blindness in adults [2]. Open-angle glaucoma (OAG), the most common form of glaucoma, includes primary open-angle glaucoma (POAG), in which intraocular pressure (IOP) is 21 mmHg or higher, and normal-tension glaucoma (NTG), in which IOP is within the normal range. NTG is common in the Japanese population, accounting for more than 90% of glaucoma cases in Japan [3], and accounts for up to 83% of OAG in China [4]. IOP-lowering therapy is the only treatment for glaucoma for which there is clinical evidence [2] and is also effective in NTG [5]. Early detection and treatment are essential in the management of glaucoma, but many patients with OAG are unaware of symptoms and do not seek medical attention until the late stages of illness [2] at a time when 25–35% of RGCs have already been lost [6]. Thus, it is important to develop objective and simple screening methods for early detection of OAG.

OAG is a multifactorial disease, and its pathogenesis is not yet fully elucidated. Elevated IOP is the greatest risk factor for the onset and progression of glaucoma. However, there are significant numbers of patients with visual field deterioration despite well-controlled IOP, suggesting that other risk factors exist for OAG progression [7]. Indeed, previous studies suggest that autoimmune mechanisms are involved in the pathogenesis of glaucoma [8,9,10]. Consistent with this, autoantibodies against heat shock proteins (HSPs) [11], γ-enolase [12], myelin basic protein (MBP) [13], and other antigens [14] have been detected in OAG.

In this study, we analyzed plasma autoantibodies using the comprehensive wet protein array (CWPA) [15,16,17,18], a type of proteome-wide analysis. We also performed multivariate receiver operating characteristic (ROC) analyses of multiple autoantibodies in combination with age, sex, and IOP to detect OAG.

## 2. Materials and Methods

### 2.1. Subjects

This study used a retrospective case–control design and was approved by the Ethics Committee of Tohoku University (2022-1-831). Patients who attended the Department of Ophthalmology of Tohoku University Hospital from March 2015 to May 2018 were included in this study after providing full written informed consent in accordance with the Declaration of Helsinki on Medical Research Involving Human Subjects. Blood tests and ophthalmologic examinations were performed. Patient information, symptoms, medications, and laboratory findings at the closest point in time to the date of blood tests were collected retrospectively from electronic medical record information.

All patients were evaluated for visual acuity, IOP, central corneal thickness (CCT), and axial length (AL), and underwent fundus photography and optical coherence tomography (OCT). The mean deviation (MD) was measured with the Humphrey Field Analyzer (HFA; Carl Zeiss Meditec, Dublin, CA, USA) using the Swedish Interactive Threshold Algorithm (SITA)-standard strategy of the 24-2 program.

Glaucoma was defined by the presence of glaucomatous optic nerve papillary changes (diffuse, focal thinning of the optic nerve limbus) and corresponding glaucomatous visual field defects. Glaucomatous optic nerve papillary changes were determined by fundus photographs and OCT. Glaucomatous visual field defects were defined by the Anderson–Patella diagnostic criteria [19] using Humphrey visual field measures:

(1) Three or more points with *p* < 5%, except for the most peripheral inspection points in the pattern deviation probability plot that are adjacent to each other with *p* < 1%.
(2) Pattern standard deviation with a probability of less than 5%.
(3) Glaucoma hemifield test indicating that the field is outside normal limits.

The OAG group included patients with mild or early (MD ≥ −6 dB; 11.8%) and progressive (MD < −6 dB; 88.2%) glaucomatous visual field loss [20]. NTG was defined as IOP ≤ 21 mmHg before treatment initiation, and POAG was defined as IOP > 21 mmHg before treatment initiation. The control group consisted of age- and sex-matched cataract patients without other ocular diseases, including glaucoma. IOP, fundus photographs, and OCT imaging tests were referenced to exclude ocular diseases other than glaucoma and cataract. Considering the average age of the OAG group, most patients already had cataracts [21,22]. Therefore, patients seen at our hospital who only had cataracts were used as the control group. The control subjects had cataracts classified as grade 2 or lower according to the Emery Little classification. OAG cases and controls were excluded if they self-reported a history of autoimmune diseases, cancers, or internal ocular surgery within the past year as these conditions could affect blood autoantibodies. Other systemic diseases were not excluded; subjects with glaucoma types other than NTG and POAG were excluded. Statistical methods were not used to predetermine the sample size as this was an exploratory analysis.

### 2.2. Wet Proteome Analysis

A proteome-wide autoantibody screening method was used as previously outlined [16,17,18]. All blood samples were allowed to stand for 30 min, centrifuged at 4 °C and 2000× *g* for 25 min, and then plasma was separated and frozen at −80 °C. The plasma was then stored at −80 °C.

#### 2.2.1. Protein Expression In Vitro

A total of 19,446 human proteins for antigens were synthesized from HuPEX (Human Proteome Expression Resource) entry clones of the entire human proteome cDNA library [15]. pEW-5FG with a SP6 promoter and an N-terminal FLAG-GST tag was used as the destination vector and protein synthesis was performed using the wheat germ cell-free translation system (CellFree Sciences Co., Ltd., Matsuyama, Japan) [15].

#### 2.2.2. Step 1. Measurement of Plasma Autoantibodies by Comprehensive Wet Protein Array (CWPA) in Initial Screening

Autoantibody measurements were performed with a previously reported CWPA [16,17,18] that contains 19,446 human proteins. A glutathione (GSH)-modified glass plate (S339009, Matsunami Glass Ind., Ltd., Kishiwada, Japan) was used for plating. Human proteins were spotted onto the plate using a HORNET-NX multi-dispense system equipped with a 1536-pin head (Fujifilm Wako Pure Chemical Co., Osaka, Japan) and bound via their GST-tags. As a preliminary study, CWPA analysis was performed using mixed plasma from each of 10 NTG, 10 POAG, and 10 cataract cases to measure autoantibodies. Then, 3:1000 diluted plasma was added to the CWPA and incubated at room temperature for 1 h. The blocking buffer described by Fukuda et al. [16] was prepared as follows: 50 mM 4-(2-hydroxyethyl)-1-piperazineethanesulfonic acid, pH 7.4, 200 mM NaCl, 1 mM DTT, 0.3% (*w*/*v*) skim milk, 5 mM GSH, 25%(*v*/*v*) glycerol, and 0.08% Triton X-100. After washing, goat anti-human IgG (H + L) Alexa Flour^®^ 647 conjugate (A21445, Thermo Fisher Scientific, Waltham, MA, USA) diluted 1:1000 was added and incubated at room temperature for 1 h. After washing again, the CWPA was air-dried and fluorescence images were measured with a fluorescence imager (Typhoon FLA 9500, Cytiva, Marlborough, MA, USA). Fluorescent images were used for quantitative analysis of autoantibodies (Supplementary Figure S1).

#### 2.2.3. Step 2. Plasma Autoantibody Assay with Custom-Designed WPA

The next step was quantification of autoantibodies in patient plasma of each disease type by custom-designed WPA. A GSH-modified glass plate (SDM0011, Matsunami Glass Industry Co., Ltd., Kishiwada, Japan) was used for custom-designed WPA. GST-tag fused human proteins were spotted using a 1536-channel independent cylinder system (EDR-384SX; Biotec, Tokyo, Japan). The target antigen of the autoantibody detected by CWPA in Step 1, previously reported glaucoma-related proteins, proteins expressed in the optic nerve, and astrocytes were spotted on custom-designed WPA. The reaction conditions and fluorescence-image-capturing method for custom-designed WPA are the same as for CWPA.

#### 2.2.4. Protein Array Data Analysis

For quantification of autoantibodies, the level of each autoantibody was calculated based on the fluorescence values obtained from the reaction of plasma with the protein spot [16]. The level of each autoantibody was calculated as follows.

$$\begin{array}{l} \text{Autoantibody titer (in arbitrary units, AUs)}\\ =\left(F_{\mathrm{autoantigen}}-F_{\mathrm{negative}\;\mathrm{controls}}\right)/\left(F_{\mathrm{positive}\;\mathrm{controls}}-F_{\mathrm{negative}\;\mathrm{controls}}\right)\times 100 \end{array}$$

F_autoantigen_: fluorescence intensity of autoantigen (human protein) spots reacted with plasma;

F_negative controls_: average fluorescence intensity of negative control (negative control for protein synthesis) spots (mock) reacting with plasma;

F_positive control_: average fluorescent intensity of positive control (human immunoglobulin to which the anti-human IgG used to detect autoantibodies binds directly).

To detect the binding of each antibody to CWPA and custom-designed WPA, positive spots were determined by the average + 3 standard deviation of the negative control spots as a threshold. As a result, for convenience, an AU value > 10 was defined as positive, and the positivity rate for each sample was calculated as follows.

Positivity rate (PR) = number of positive samples/number of samples in disease group × 100

### 2.3. Statistical Analysis

All analyses were performed using R version 4.1.2. In addition, *t*-tests and Fisher’s exact test were used to compare the backgrounds of the subjects. A comparison of autoantibody titers in each form was carried out using Dunnett’s multiple comparisons test, and the association of each autoantibody in OAG with age, sex, and ophthalmic clinical information (MD, IOP, AL, CCT) was evaluated using Spearman’s correlation coefficient. The association of each autoantibody was also evaluated using Spearman’s correlation coefficient. For ophthalmologic examination items, the visual field severity eye was employed in OAG, while the right eye was employed in the control cataract group. To evaluate the diagnostic power of each autoantibody in each disease type, ROC curves were drawn using sensitivity and specificity calculated from cross-tabulations, and optimal cutoff and the area under the curve (AUC) values were obtained.

It should be noted that this criterion for positivity by optimal cutoff value differs from the criterion that defines an AU value > 10 as positive. This was carried out in order to detect binding of each antibody to CWPA and custom-designed WPA. A comparison of the positivity rates in each disease group was performed by Fisher’s multiple testing (with Bonferroni correction to account for multiple comparisons).

To evaluate the diagnostic power of multiple autoantibodies, we used all glaucoma autoantibodies corrected for age, sex, and IOP to draw ROC curves and obtain AUC values by logistic regression analysis. Model 1 consisted of age, sex, and IOP variables. Model 2 consisted of age, sex, IOP, and all four autoantibody variables. The AUC for each model was tested with the Delong test. For all analyses (except Fisher’s multiple testing for comparing multiple antibodies), p values were considered statistically significant, when the values were lower than 0.05 (two-tailed).

## 3. Results

Initial screening identified 171 antigens, 149 of which tested positive only for OAG and 22 of which tested positive only for cataracts. These 171 antigens, plus 25 antigens previously associated with glaucoma and 24 antigens expressed in the optic nerve and astrocytes, leading to a total of 220 antigens, were loaded onto custom-designed WPA plates (Supplementary Table S1) and analyzed for plasma autoantibodies in cataract, NTG, and POAG. In total, 35 cataract, 50 NTG, and 69 POAG samples were available for study, and 99 antigens were detectable from these samples. Table 1 shows the clinical characteristics of the patients from whom these specimens were obtained. Age, sex, IOP, and CCT showed no significant differences. Among the OAG group, IOP was 12.67 ± 2.54 mmHg in the NTG group and 15.89 ± 4.99 mmHg in the POAG group (*p* < 0.001). MD was −17.50 ± 7.98 dB in the NTG group and −19.27 ± 8.93 dB in the POAG group (no significant difference between the two groups, *p* = 0.268). Most OAG patients (88%) were in an advanced stage, with few cases (12%) better than −6 dB. AL showed a significant difference between the cataract and OAG groups (*p* = 0.042).

### 3.1. Extraction of Autoantibodies

**Figure 1:**
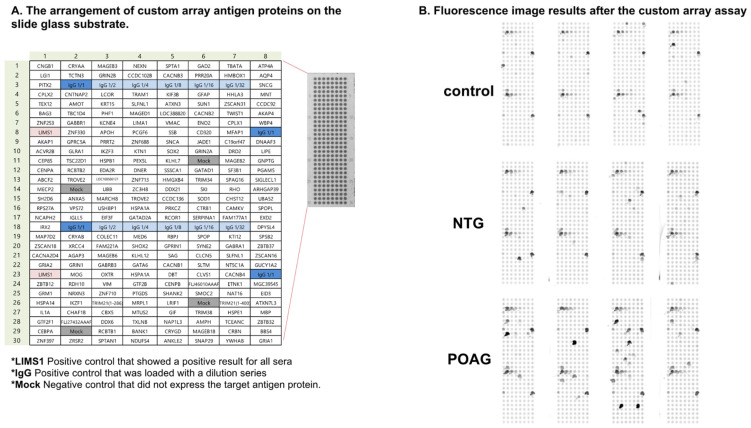
The custom-designed WPA. (**A**). The panel depicts the arrangement of custom array antigen proteins on the slide glass substrate. *LIMS1 was a positive control that showed positive results for all sera. *IgG was also a positive control that was loaded with a dilution series. *Mock was a negative control that did not express the target antigen protein. (**B**). Fluorescence image results after the custom array assay are shown.

**Figure 2:**
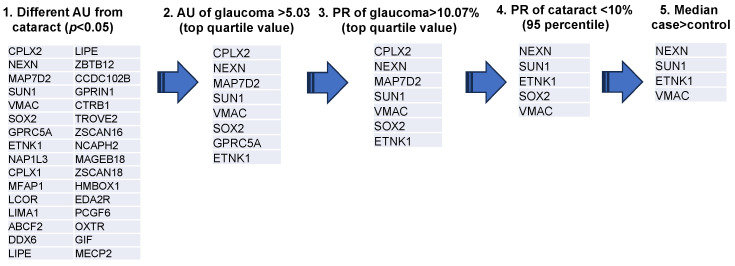
Extraction process of glaucoma associated autoantibodies. The diagram depicts the procedure for selection of glaucoma-associated autoantibodies. Step 1 was to extract 32 antigens for which there was a statistically significant difference in AUs between cataract and glaucoma groups (OAG, NTG, and POAG). Step 2 screened eight antigens with antibody titers in the top quartile. Step 3 screened seven antigens with positive titers in the top quartile for AU. Step 4 excluded two antibodies that fell into the top 95% of positivity rate for cataracts. Step 5 excluded SOX2, which showed higher median values in the cataract group compared to the glaucoma group. Antigen proteins targeted by autoantibodies were described by the gene symbol from which they were derived.

A representative example of the custom-designed WPA results is shown in Figure 1. From these results, the AUs (antibody titer in arbitrary units) and PR were calculated for each disease for the 99 autoantigens detected in the custom-designed WPA (Supplementary Table S2). We next extracted 32 glaucoma-related autoantibodies that showed statistically significant differences in titers between the cataract and glaucoma groups (Figure 2). Among them, we selected the top quartile of antibody titers in the glaucoma group (AU > 5.03) and the top quartile of positivity rates (PR > 10.07). Two antibodies, showing high titers and positivity rates for cataracts (in the top 95% of the samples), were excluded. Finally, SOX2, which has a higher median in the cataract group than the glaucoma group, was excluded. As a result, ethanolamine kinase 1 (ETNK1), vimentin-type intermediate filament-associated coiled-coil protein (VMAC), nexilin (NEXN), and Sad1 and UNC84 Domain Containing 1 (SUN1) were identified as glaucoma-associated autoantibodies.

### 3.2. Comparison of Autoantibody Titers

**Figure 3:**
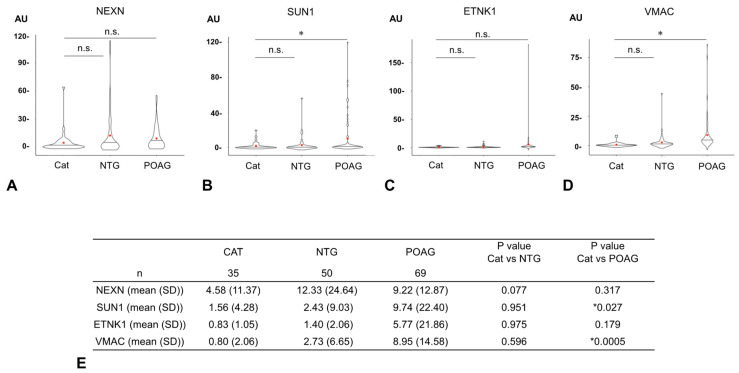
Comparison of autoantibody titers between cataract and glaucoma. The graphs show violin plot analyses comparing the distribution of antibody titers (AUs) of each glaucoma-related autoantibody by disease type ((**A**). NEXN, (**B**). SUN1, (**C**). ETNK, (**D**). VMAC). The antibody titers of autoantibodies between the cataract (CAT), NTG, and POAG groups were compared by Dunnett’s multiple comparison test (* *p* < 0.05) and are summarized in table (**E**). The n.s. indicates not significant. The red dot represents the average value. For SUN1 and VMAC, autoantibody titers (AUs) were significantly higher in the POAG group than in the cataract group, while there were no significant differences between the cataract and NTG groups. For other autoantibodies (NEXN, ETNK), there were no significant differences in AUs between the cataract and NTG groups or between the cataract and POAG groups.

The AUs of all four glaucoma-related autoantibodies were compared for the 35 cataract, 50 NTG, and 69 POAG samples (Figure 3). For SUN1 and VMAC, the AUs were significantly higher in the POAG group than in the cataract group, but no significant difference was detected between the cataract and NTG groups. For other autoantibodies (NEXN, ETNK1), there were no significant differences in AUs between the cataract and NTG groups or between the cataract and POAG groups.

### 3.3. Examination of the Ability of Each Autoantibody to Discriminate Between the Cataract and Glaucoma Groups

**Figure 4:**
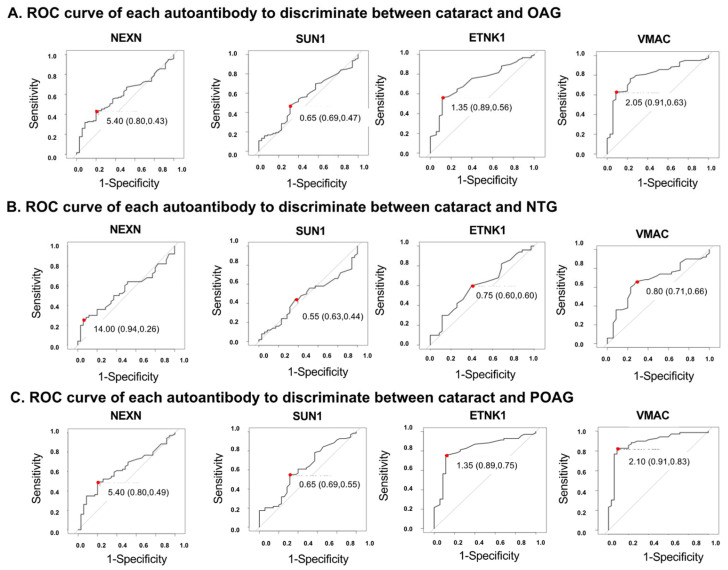
ROC curves for the ability of each autoantibody to discriminate between cataracts and OAG. Optimal cutoff values are indicated by red dots. Optimal cutoff value, sensitivity, and specificity, shown in parentheses, are inserted in each graph. (**A**). ROC curve for each autoantibody when discriminating OAG from cataract. Optimal cutoff value, sensitivity, and specificity was 5.40 (0.80, 0.43) in NEXN, 0.65 (0.69, 0.47) in SUN1, 1.35 (0.89, 0.56) in ETNK1, 2.05 (0.91, 0.63) in VMAC. (**B**). ROC curve for each autoantibody when discriminating NTG from cataract. Optimal cutoff value, sensitivity, and specificity was 14.00 (0.94, 0.26) in NEXN, 0.55 (0.63, 0.44) in SUN1, 0.75 (0.60, 0.60) in ETNK1, 0.80 (0.71, 0.66) in VMAC. (**C**). ROC curve for each autoantibody when discriminating POAG from cataract. Optimal cutoff value, sensitivity, and specificity was 5.40 (0.80, 0.49) in NEXN, 0.65 (0.69, 0.55) in SUN1, 1.35 (0.89, 0.75) in ETNK1, 2.10 (0.91, 0.83) in VMAC.

An ROC analysis was performed for each autoantibody to discriminate the glaucoma groups from the cataract (control) group (Figure 4, Supplementary Table S3). To determine the optimal cutoff values, a Youden Index, defined as sensitivity-(1-specificity), was calculated. The AU with the largest Youden Index was used as the optimal cutoff value (Supplementary Figure S2). For example, in the ROC analysis of the discrimination between cataract and POAG groups using ETNK1 (Supplementary Table S3), the autoantibody titer with the maximum Youden Index is 1.35. This is the optimal cutoff value to separate cataract and POAG. In this case, sensitivity is 0.886, specificity is 0.754, the Youden Index is 0.64.

In discriminating between the OAG and cataract (control) groups (Figure 4A, Supplementary Table S3), autoantibodies against ETNK1 and VMAC were highest among the four autoantibodies, with AUCs of 0.728 (95%CI: 0.639–0.817) and 0.796 (95%CI: 0.717–0.876), respectively. VMAC showed the highest AUC (0.669 [95%CI: 0.615–0.832]) to make a distinction between NTG and cataract (Figure 4B, Supplementary Table S3). In discrimination between the POAG and cataract groups (Figure 4C, Supplementary Table S3), the AUCs of ETNK1 and VMAC were calculated to be 0.820 (95%CI: 0.733–0.907) and 0.889 (95%CI: 0.818–0.959), respectively.

### 3.4. Comparison of Autoantibody Positivity Rates

**Figure 5:**
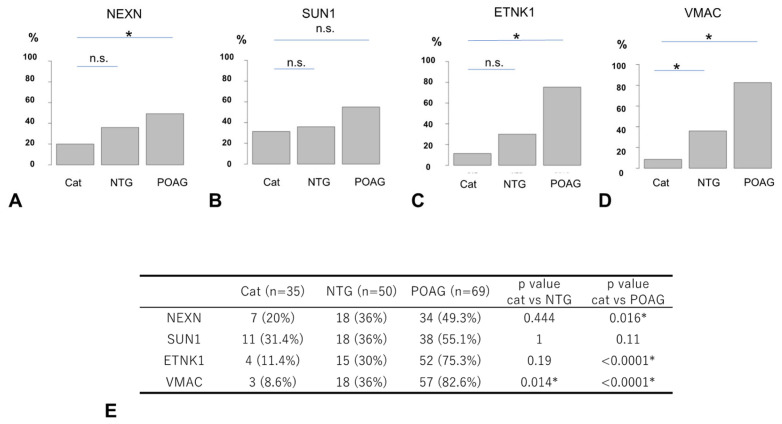
Comparison of positivity rates between cataract and glaucoma. Percentage positive for each autoantibody using the optimal cutoff value for OAG as a threshold ((**A**). NEXN, (**B**). SUN1, (**C**). ETNK1, (**D**). VMAC). The positivity rate (PR) of autoantibodies between the cataract, NTG, and POAG groups is summarized in table (**E**) and compared by Fisher’s exact test and Bonferroni correction (* *p* < 0.05). The n.s. indicates not significant. NEXN and ETNK1 showed significant differences in the PR between cataracts and POAG, but not between cataracts and NTG. VMAC showed significant differences in the PR between cataracts and POAG and between cataracts and NTG. SUN1 showed no significant difference in the PR between cataract and POAG and between cataract and NTG.

The comparison of the positivity rate (PR) by disease type for each autoantibody is shown in Figure 5, where positive and negative results were determined based on the optimal cutoff value for discriminating between the OAG and cataract groups (Figure 4, Supplementary Table S3). The PR of antibodies to NEXN and ETNK1 was significantly higher in POAG compared to cataract, while there was no significant difference between NTG and cataract (Figure 5). For antibodies to SUN1, there was no significant difference in the PR between the different types of glaucoma and cataract. The PR of antibodies to VMAC was significantly higher in the NTG and POAG groups compared to the cataract group.

### 3.5. Overlap of Autoantibodies

Examination of overlap of autoantibody in NTG and POAG revealed that in NTG, autoantibodies showed overlap in 48.8% of cases. On the other hand, POAG had clearly more cases of autoantibody overlap than NTG with 88.2% (Supplementary Figure S3). Among the overlapping antibodies, those containing NEXN were the most common in NTG (65.0%) and those containing VMAC were the most common in POAG (91.5%).

### 3.6. Relationship Between Autoantibodies and Clinical Findings

Spearman’s rank correlation coefficient test showed that autoantibodies against ETNK1, VMAC, NEXN, or SUN1 were not significantly correlated with each other (Supplementary Table S4). In all glaucoma types, the four glaucoma-associated autoantibodies other than NEXN did not correlate significantly with any of the clinical factors: MD, IOP, IOL AL, CCT, or age (Table 2). The NEXN autoantibody correlated with age only in POAG, but did not correlate significantly with MD, IOP, AL, or CCT. To explore the usefulness of each antibody in the detection of early-stage glaucoma, we compared the positivity rates between early-stage and more-progressed-stage glaucoma. No significant differences between these two stages were observed for any of the antibodies (Supplementary Figure S4).

### 3.7. Logistic Regression Analysis to Predict Glaucoma Using a Set of Glaucoma-Associated Autoantibodies

**Figure 6:**
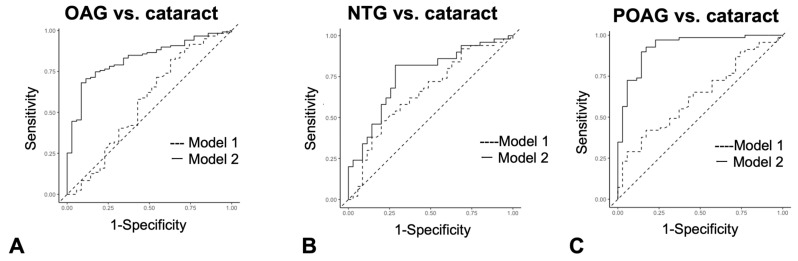
Discrimination between cataract and glaucoma by the ROC logistic regression model. (**A**–**C**). ROC logistic regression model for glaucoma discrimination. Model 1 consists of age, sex, and IOP variables. Model 2 consists of variables for age, sex, IOP and all four autoantibodies. (**A**). ROC logistic regression analysis when discriminating OAG from cataract. (**B**). ROC logistic regression analysis when discriminating NTG from cataract. (**C**). ROC logistic regression analysis when discriminating POAG from cataract.

Logistic regression analysis was used to determine if a series of autoantibody combinations were useful as diagnostic tests in discriminating OAG, NTG, and POAG from cataracts (Figure 6A–C, Supplementary Table S5). The cutoff value in logistic regression analysis is a probability and is different from antibody titer. As a result, in the comparison between cataract and OAG (Figure 6A) and cataract and POAG (Figure 6C), the AUC of Model 2, which added autoantibodies to age, sex, and IOP, was significantly greater than that of Model 1, which consisted of conventional health examination items, namely age, sex, and IOP variables. On the other hand, between cataract and NTG, the alpha level between Model 1 and Model 2 was 0.05, and the AUC difference did not reach statistical significance (Figure 6B).

## 4. Discussion

In this study, a cohort of cataract, NTG, and POAG patients was examined by CWPA for plasma autoantibodies, and four new autoantibodies, ETNK1, VMAC, NEXN, and SUN1, were detected as glaucoma-related. For each autoantibody, an ROC curve analysis was performed to examine the ability to discriminate glaucoma patients from cataract patients, and optimal cutoff values were calculated to determine the positivity rate by disease type. Furthermore, we performed multivariate analyses using all four autoantibodies to clarify the diagnostic usefulness of combining these autoantibodies compared to analyses using individual autoantibodies.

In recent years, various methods of hematological proteome-wide analysis have been developed to quantify proteins in a comprehensive manner. The WPA method used in this study has several advantages over conventional human protein array or ELISA methods. First, the CWPA plate carries 19,446 proteins for antigens that were comprehensively synthesized from a human cDNA library, and the range of self-antigens that can be loaded is comparable to the level of the entire proteome [15,16,17]. Second, the CWPA has an extremely narrow average protein loading range (Max/Min) of 47.3, making it less prone to mass bias in the antigen–antibody reaction due to protein loading. Third, the entire process is designed to maintain the protein’s three-dimensional conformation by handling the arrays under wet conditions, allowing for more accurate analysis of specific antigen–antibody binding [16,17,18]. In this study, we were unable to confirm previously reported glaucoma-associated autoantibodies such as HSPs [11], γ-enolase [12] and MBP [13]. Their original detection was mainly based on results of ELISA, which does not preserve a three-dimensional protein structure. Additionally, the sample size may influence the present results. Therefore, further investigation in a larger population is needed in the future.

Antibodies are primarily a defense system acquired to protect the body from foreign bacteria and viruses. However, antibodies are generated not only against foreign antigens, but also against proteins that are produced in excess or released outside the cells in diseases. In autoimmune diseases, autoantibodies can be both disease markers and causes of disease. In the present study, the four novel autoantibodies were not significantly correlated with the severity of visual field changes (MD value) (Table 2), indicating that these four autoantibodies are not likely to be markers of glaucoma severity.

Overlapping autoantibodies were detected in only 48.8% of patients in NTG, whereas overlap was 88.2% in POAG (Supplementary Figure S3). This finding suggests that autoimmune-positive NTG can be discriminated from autoimmune-negative NTG and each of the four autoantibodies may define a new independent entity. Also, POAG can be divided into autoantibody-related POAG (APOAG) and non-APOAG (Supplementary Figure S5).

The antigens of the four autoantibodies associated with glaucoma are all proteins implicated in the pathogenesis of glaucoma. Some of these proteins are also related to myopia, a risk factor of glaucoma, especially NTG.

NEXN is an actin-binding protein that regulates actin filament polymerization. Chronically elevated IOP induces polymerization of actin filaments in trabecular cells [23]. Although the detailed mechanism linking increased IOP and actin remodeling has not been fully elucidated, NEXN may protect trabecular cells or RGCs from the mechanical stress caused by increased IOP by promoting actin polymerization. If NEXN is released from trabecular cells or RGCs by elevated IOP in an early stage of OAG, NEXN may contribute to the pathogenesis of glaucoma in a previously uncharacterized manner.

ETNK1 is involved in the phosphorylation of ethanolamine (Et) to phosphoethanolamine (P-Et), which plays a key role in the major metabolic pathway that synthesizes phosphatidylethanolamine (PE) and phosphatidylcholine (PC), the two most abundant phospholipids in mammalian cell membranes. PE is required for optimal mitochondrial respiratory activity and ubiquinone function [24], and mitochondrial dysfunction is associated with glaucomatous neurodegeneration [25]. Furthermore, aging may reduce mitochondrial function and affect the survival of RGCs in glaucoma [26]. Thus, ENTK1 may be strongly involved in the pathophysiology of glaucoma via multiple actions, including effects on mitochondrial function.

VMAC is indirectly associated with vimentin-type intermediate filaments via vimentin-binding proteins [27]. The presence of autoantibodies to vimentin has been previously reported in patients with NTG. Since elevated IOP increases vimentin synthesis in astrocytes [28], VMAC may be associated with glaucomatous damage caused by elevated IOP. Furthermore, myopia is a known risk factor for glaucoma [29]. Myopia is characterized by prolonged ocular AL and extracellular matrix (ECM) remodeling of the sclera. Because myopia is associated with differentiation of myofibroblasts that are characterized with vimentin [30], it is plausible that VMAC contributes to the pathogenesis of glaucoma via myopia.

SUN1 is a nuclear membrane protein with an Unc84 (SUN) domain. Ueda et al. [31]. examined the effect of SUN protein deletion on maturation of the actin cytoskeleton and newly generated focal adhesions (nascent focal adhesions, FAs). SUN1 is essential for actin formation, generation of intercellular traction, and maturation of FAs [32]. SUN1 is also a key component of actin and FA maturation. Thus, SUN1 may be involved in the pathogenesis of glaucoma via actin and FAs. In addition, actin, the molecular target of SUN1, is closely related to the induction of myopia through ocular AL elongation [33,34].

In POAG, damage to the cell membrane caused by elevated intraocular pressure may have resulted in these proteins involved with the cytoskeleton and mitochondria flowing out of the cell, triggering an immune mechanism and producing autoantibodies. NTG, on the other hand, is pathologically affected by a variety of non-intraocular pressure-dependent factors, such as blood flow, neurotrophic factors, or oxidative stress [35,36,37]. As a result, there is less damage to the cell membrane due to IOP, less extravasation of these intracellular proteins, and possibly less antibody production as a result.

The present analysis has several limitations. First, the sample size is limited. Statistical methods were not used to predetermine the sample size, as this was an exploratory analysis. Considering the number of possible autoantibodies with CWPA and the sample size, there may be other autoantibodies that were not detected. This also may lead to overestimation and a decrease in the diagnostic power of these autoantibodies in OAG patients. Further investigation in a larger population is needed in the future. Second, there may be selection bias based on clinical ascertainment of the study population. The current results are based on patients from the same clinical center, and there is a risk of overfitting. To enhance generalizability, we plan to examine external validity in other cohorts. Third, although patients with autoimmune diseases or cancers that could affect the biomarkers under study were excluded, other confounding factors could have affected plasma biomarker values.

## 5. Conclusions

In the present study, four new autoantibodies have been identified in association with NTG and POAG by the application of a newly developed proteome-wide analysis. A combination of these autoantibodies appears to be a suitable biomarker for the diagnosis of OAG, even in an early disease stage. Autoantibody testing could help to promote early OAG detection and prevent severe vision loss by timelier onset of treatment.

## 6. Patents

Tohoku University, Proteobridge, Santen Pharmaceutical Co., Ltd., Diagnosis of glaucoma, P2023-129215A, Japan Patent Office, JPO, 2023.

## Figures and Tables

**Table 1:** Characteristics of each study population.

	Cat		OAG		*p* Value cat vs. OAG	*p* Value NTG vs. POAG
		Total	NTG	POAG		
N	35	119	50	69		
Age (mean (SD))	69.97 (10.84)	68.76 (7.03)	68.48 (6.04)	68.96 (7.71)	0.433	0.717
Sex = Male (%)	18 (51.4)	68 (57.1)	25 (50.0)	43 (62.3)	0.686	0.249
IOP (mean (SD))	14.06 (3.31)	14.54 (4.43)	12.67 (2.54)	15.89 (4.99)	0.553	<0.001 *
MD (mean (SD))			−17.50 (7.98)	−19.27 (8.93)		0.268
AL (mean (SD))	24.50 (2.29)	25.24 (1.64)	25.15 (1.45)	25.31 (1.77)	0.042 *	0.616
CCT (mean (SD))	509.00 (34.07)	502.03 (35.83)	494.46 (32.07)	507.52 (37.61)	0.332	0.049*

The clinical characteristics of the patients from whom specimens were obtained were statistically compared with each other as described in the Section 2. * *p* < 0.05.

**Table 2:** Spearman’s rank correlation of each autoantibody with age, IOP, and ophthalmic clinical parameters.

OAG				
	ETNK1	VMAC	NEXN	SUN1
MD	0.057	−0.020	−0.182	−0.011
IOP	0.095	0.128	−0.022	−0.005
AL	−0.045	−0.042	0.073	0.027
CCT	0.167	0.043	0.008	0.0117
Age	−0.035	−0.066	0.183	0.001
**NTG**				
	ETNK1	VMAC	NEXN	SUN1
MD	0.057	−0.020	−0.182	−0.011
IOP	0.095	0.128	−0.022	−0.005
AL	−0.045	−0.042	0.073	0.027
CCT	0.167	0.043	0.008	0.011
Age	−0.035	−0.066	0.183	0.001
**POAG**				
	ETNK1	VMAC	NEXN	SUN1
MD	0.234	0.068	−0.168	0.104
IOP	−0.178	−0.098	−0.033	−0.205
AL	−0.079	−0.040	0.038	−0.006
CCT	0.123	0.055	0.015	0.112
Age	−0.187	−0.094	0.273 *	−0.077

* *p* < 0.05.

## Data Availability

Restrictions apply to the datasets. The datasets presented in this article are not readily available because the data are part of an ongoing study. Requests to access the datasets should be directed to the corresponding author(s).

## Supplementary Materials

The following supporting information can be downloaded at: https://www.mdpi.com/article/10.3390/biomedicines13030718/s1, Table S1: Antigen proteins on custom arrays. References [38,39,40,41,42,43,44,45,46,47,48,49,50,51,52,53,54,55,56,57,58,59,60,61,62,63,64,65,66,67,68,69] are cited in the supplementary materials.; Table S2: Antibody titers and positivity rates by disease type for 99 autoantibodies that could be detected by custom arrays; Table S3: ROC analysis of each disease type; Table S4: Spearman’s rank correlation between autoantibodies in OAG; Table S5: ROC logistic analysis between glaucoma and cataract; Figure S1: Representative image of proteins bound on the CWPA; Figure S2: Relationship between optimal cutoff value by Youden Index, sensitivity, and specificity; Figure S3: Autoantibody overlap in NTG (A) and POAG (B); Figure S4: Comparison of positivity rates between early stage and progress stage of glaucoma. Figure S5. Diagnostic criteria for glaucoma.
