# Supplementary material for: Proteome-Wide Analysis of Autoantibodies in Open-Angle Glaucoma in Japanese Population: A Pilot Study
Source: Biomedicines. 2025 Mar 14;13(3):718. doi: 10.3390/biomedicines13030718 (PMC11940370; doi:10.3390/biomedicines13030718)
Supplement: Supplementary file 1 [file biomedicines-13-00718-s001.zip › biomedicines-3485721-supplementary.pdf]

Supporting Table 1. Antigen proteins on custom arrays.

Group	Number	Antigen
previously reported glaucoma associated autoantibodies	25	Glial fibrillary acidic protein (GFAP)[38], Vimentin[39], Isoform1 of HSP70[39], HSP70[39], HSP60[40], HSP27[40], HSP10[41], β-L-Crystallin[39], αB-Crystallin[42], 14-3-3 protein β/α[43], α-crystallin A chain[44] ,Annexin A5[44], Myelin basic protein (MBP)[45], Superoxide dismutase[46], γ-enolase[47], Retinal S-antigen[48], β2-glycoprotein[49], γ-synuclein[38], α-synuclein[50], Ubiquitin（3 types)[51], α-1-antitrypsin[52], Rhodopsin[53], a-fodrin[54]
Glaucoma-specific antigens in initial autoantibody profiling in a discovery sample set	149	TRIM21(1-286), TRIM21(1-400), GIF, SSB, LIMS1, TROVE2, RCBTB1, SPTA1, CEP85, SPSB2, TBC1D4, DBT, KLHL7, SLTM, TRIM38, TROVE2, ZNF330, ZNF688, CPLX1, NAP1L3, RBPJ, ZRSR2, CPLX2, GATAD2A, KIF3B, ZBTB32, AGAP3, AMOT, FLJ46010AAAF, IL1A, MAGED1, NEXN, SNAP29, SOX2, SUN1, TWIST1, KLHL12, VMAC, KRT15, MECP2, PRRT2, SHOX2, TBATA, ABCF2, ARHGAP39, ATP4A, ATXN3, BANK1, C19orf47, CCDC102B, CCDC136, CCDC92, CEBPA, CHST12, CRBN, DDX21, DDX6, DNAAF3, DPYSL4, EDA2R, EID3, FLJ27432AAAF, GATA6, GATAD1, GNPTG, GPRC5A, GTF2B, GTF2F1, GUCY1A2, HHLA3, IKZF1, IKZF3, JADE1, LCOR, LIMA1, LOC100506127, LRIF1, MAGEB18, MAGEB2, MAGEB3, MAGEB6, MAP7D2, MED6, MFAP1, MRPL1, OXTR, PHF1, PITX2, PTGDS, RCBTB2, RDH10, SHANK2, SKI, SMOC2, SSSCA1, TRIM34, TXLNB, ZBTB12, ZNF253, ZNF710, ZNF713, ZSCAN16, ZSCAN18, ZSCAN31, ETNK1, FAM221A, FAM177A1, NAT16, SIGLECL1, BBS4, EIF3F, ZNF397, ACVR2B, AKAP4, ANKLE2, CD320, CHAF1B, CLCN5, CLVS1, COLEC11, CTRB1, EXD2, GPRIN1, HMBOX1, HMGXB4, KTI12, KTN1, LIPE, LOC388820, MGC39545, MNT, NCAPH2, NT5C1A, PCGF6, PEX5L, PGAM5, PRR20A, RCOR1, SF3B1, SH2D6, SLFNL1, SLFNL1, TCEANC, TCTN3, TEX12, TRAM1, USHBP1, VPS72, WBP4, ZC3H8
Cataract-specific antigens in initial autoantibody profiling in a discovery sample set	22	SPOP, CENPA, CENPB, ATXN7L3, NDUFS4, CBX5, IGLL5, MARCH8, PRKCZ, SPAG16, SPOPL, AKAP1, BAG3, CAMKV, CNGB1, IRX2, KCNE4, MTUS2, SYNE2, TSC22D1, XRCC4, ZBTB37,
Antibodies expressed in optic nerve fiber or astrocyte	24	NMDAR (subunit 1, 2B, 2A)[55], AMPAR (subunit 1, 2)[56], LGI1[57], AQP4[58], MOG[59], GAD65[60], GABAAR (subunit β3, α1)[61], GABABR (subunit 1)[62], CASPER2[63], DRD2[64], Neuroxin-3α[65], DNER[66], P/Q-type VGCC (subunit β2, β4, β1, β3, α2δ4)[67], mGluR1[68], Glycine receptor[69], Amphiphysin[70]
Total antigen	220	

References

1. Von Thun Und Hohenstein-Blaul, N.; Kunst, S.; Pfeiffer, N.; Grus, F.H. Biomarkers for glaucoma: From the lab to the clinic. *Eye* **2017**, *31*, 225–231. https://doi.org/10.1038/eye.2016.300.
2. Joachim, S.C.; Bruns, K.; Lackner, K.J.; Pfeiffer, N.; Grus, F.H. Antibodies to alpha B-crystallin, vimentin, and heat shock protein 70 in patients with normal tension glaucoma and IgG antibody patterns against retinal antigen in aqueous humor. *Curr. Eye Res.* **2007**, *32*, 501–509.
3. Tsai, T.; Grotegut, P.; Reinehr, S.; Joachim, S.C. Role of Heat Shock Proteins in Glaucoma. *Int. J. Mol. Sci.* **2019**, *20*, 5160. https://doi.org/10.3390/ijms20205160.
4. Vanags, D.; Williams, B.; Johnson, B.; Hall, S.; Nash, P.; Taylor, Al.; Weiss, J.; Feeney, D. Therapeutic efficacy and safety of chaperonin 10 in patients with rheumatoid arthritis: A double-blind randomised trial. *Lancet* **2006**, *368*, 855–863. https://doi.org/10.1016/S0140-6736(06)69210-6.
5. Mirzaei, M.; Gupta, V.B.; Chick, J.M.; Greco, T.M.; Wu, Y.; Chitranshi, N.; Wall, R.V.; Hone, E.; Deng, L.; Dheer, Y.; et al. Age-related neurodegenerative disease associated pathways identified in retinal and vitreous proteome from human glaucoma eyes. *Sci. Rep.* **2017**, *7*, 12685. https://doi.org/10.1038/s41598-017-12858-7.
6. Bell, K.; Wilding, C.; Funke, S.; Pfeiffer, N.; Grus, F.H. Protective effect of 14-3-3 antibodies on stressed neuroretinal cells via the mitochondrial apoptosis pathway. *BMC Ophthalmol.* **2015**, *15*, 64. https://doi.org/10.1186/s12886-015-0044-9.
7. Yap, T.E.; Davis, B.M.; Guo, L.; Normando, E.M.; Cordeiro, M.F. Annexins in Glaucoma. *Int. J. Mol. Sci.* **2018**, *19*, 1218. https://doi.org/10.3390/ijms19041218.
8. Joachim, S.C.; Reichelt, J.; Berneiser, S.; Pfeiffer, N.; Grus, F.H. Sera of glaucoma patients show autoantibodies against myelin basic protein and complex autoantibody profiles against human optic nerve antigens. *Graefes Arch. Clin. Exp. Ophthalmol.* **2008**, *246*, 573–580. https://doi.org/10.1007/s00417-007-0737-8.
9. Beutgen, V.M.; Perumal, N.; Pfeiffer, N.; Grus, F.H. Autoantibody biomarker discovery in primary open angle glaucoma using serological proteome analysis (SERPA). *Front. Immunol.* **2019**, *10*, 381. https://doi.org/10.3389/fimmu.2019.00381.

10. Maruyama, I.; Ohguro, H.; Ikeda, Y. Retinal ganglion cells recognized by serum autoantibody against gamma-enolase found in glaucoma patients. *Invest. Ophthalmol. Vis. Sci.* **2000**, *41*, 1657–1665.
11. Boehm, N.; Wolters, D.; Thiel, U.; Lossbrand, U.; Wietgel, alN.; Pfeiffer, N.; Grus, F.H. New insights into autoantibody profiles from immune privileged sites in the eye: A glaucoma study. *Brain Behav. Immun.* **2012**, *26*, 96–102. https://doi.org/10.1016/j.bbi.2011.07.241.
12. Latalska M, Gerkowicz M, Kosior-Jarecka E, Kozioł-Montewka M, Pietraś-Trzpiel, M. Antibodies to beta-2 glycoprotein I in serum and aqueous humor of patients with glaucoma and their influence on the static perimetry. *Klin. Oczna* **2004**, *106*, 160–161.
13. Surgucheva, I.; McMahan, B.; Ahmed, F.; Tomarev, S.; Wax, M.B.; Surguchov, A. Synucleins in glaucoma: Implication of gamma-synuclein in glaucomatous alterations in the optic nerve. *J. Neurosci. Res.* **2002**, *68*, 97–106. https://doi.org/10.1002/jnr.10198.
14. Saccà, S.C.; Centofanti, M.; Izzotti, A. New proteins as vascular biomarkers in primary open angle glaucomatous aqueous humor. *Invest. Ophthalmol. Vis. Sci.* **2012**, *53*, 4242–4253. https://doi.org/10.1167/iovs.11-8902.
15. Romano, C.; Barrett, D.A.; Li, Z.; Pestronk, A.; Wax, M.B. Anti-rhodopsin antibodies in sera from patients with normal-pressure glaucoma. *Invest. Ophthalmol. Vis. Sci.* **1995**, *36*, 1968–1975.
16. Grus, F.H.; Joachim, S.C.; Bruns, K.; Lackner, K.J.; Pfeiffer, N.; Wax, M.B. Serum autoantibodies to alpha-fodrin are present in glaucoma patients from Germany and the United States. *Invest. Opthalmol Vis. Sci.* **2006**, *47*, 968–976.
17. Yoshii, A.; Sheng, M.H.; Constantine-Paton, M. Eye opening induces a rapid dendritic localization of PSD-95 in central visual neurons. *Proc. Natl. Acad. Sci. USA* **2003**, *100*, 1334–1339. https://doi.org/10.1073/pnas.0335785100.
18. García-Barcina, J.M.; Matute, C. AMPA-selective glutamate receptor subunits in glial cells of adult bovine white matter. *Brain Res. Mol. Brain Res.* **1998**, *53*, 270–276. https://doi.org/10.1016/s0169-328x(97)00318-5.
19. Zhou, Y.D.; Zhang, D.; Ozkaynak, E.; Wang, X.; Kasper, E.M.; Leguern, E.; Baulac, S.; Anderson, M.P. Epilepsy gene LGI1 regulates postnatal developmental remodeling of retinogeniculate synapses. *J. Neurosci.* **2012**, *32*, 903–910. https://doi.org/10.1523/JNEUROSCI.5191-11.2012.
20. Lennon, V.A.; Wingerchuk, D.M.; Kryzer, T.J.; Pittock, S.J.; Lucchinetti, C.F.; Fujihara, K.; Nakashima, I.; Weinshenker, B.G. A serum autoantibody marker of neuromyelitis optica: Distinction from multiple sclerosis. *Lancet* **2004**, *364*, 2106–2112. https://doi.org/10.1016/S0140-6736(04)17551-X.
21. Kezuka, T.; Ishikawa, H. Diagnosis and treatment of anti-myelin oligodendrocyte glycoprotein antibody positive optic neuritis. *Jpn. J. Ophthalmol.* **2018**, *62*, 101–108. https://doi.org/10.1007/s10384-018-0561-1.
22. Yasin, A.; Dudeck, L.; Redick, D.W.; Khodeiry, M.M.; Lam, B.L.; Jiang, H. Severe vision loss and optic disc edema associated with GAD-65 antibody positive Miller Fisher syndrome. *J. Neuroophthalmol.* **2022**, *44*, e40–e44. https://doi.org/10.1097/WNO.
23. Huntsman, M.M.; Jones, E.G. Expression of alpha3, beta3 and gamma1 GABA(A) receptor subunit messenger RNAs in visual cortex and lateral geniculate nucleus of normal and monocularly deprived monkeys. *Neuroscience* **1998**, *87*, 385–400. https://doi.org/10.1016/s0306-4522(98)00140-7.
24. Moldavan, M.G.; Allen, C.N. GABAB receptor-mediated frequency-dependent and circadian changes in synaptic plasticity modulate retinal input to the suprachiasmatic nucleus. *J. Physiol.* **2013**, *591*, 2475–24390. https://doi.org/10.1113/jphysiol.2012.248047.
25. Qin, X.; Yang, H.; Zhu, F.; Wang, Q.; Shan, W. Clinical character of CASPR2 autoimmune encephalitis: A multiple center retrospective study. *Front. Immunol.* **2021**, *12*, 652864. https://doi.org/10.3389/fimmu.2021.652864.

26. Yang, Q.; Jiang, M.; Xu, S.; Yang, L.; Yang, P.; Song, Y.; Zhu, H.; Wang, Y.; Sun, Y.; Yan, C.; et al. Mirror image pain mediated by D2 receptor regulation of astrocytic Cx43 phosphorylation and channel opening. *Biochim. Biophys. Acta Mol. Basis Dis.* **2023**, *1869*, 166657. https://doi.org/10.1016/j.bbadis.2023.166657.
27. Bhat, M.A.; Rios, J.C.; Lu, Y.; Garcia-Fresco, G.P.; Ching, W.; Martin, M.S.; Li, J.; Einheber, S.; Chesler, M.; Rosenbluth, J.; et al. Axon-glia interactions and the domain organization of myelinated axons requires neurexin IV/Caspr/Paranodin. *Neuron* **2001**, *30*, 369–383. https://doi.org/10.1016/s0896-6273(01). 00294-x.
28. Keeley, P.W.; Reese, B.E. DNER and NFIA are expressed by developing and mature AII amacrine cells in the mouse retina. *J. Comp. Neurol.* **2018**, *526*, 467–479. https://doi.org/10.1002/cne.24345.
29. Murali, S.S.; Napier, I.A.; Mohammadi, S.A.; Alewood, P.F.; Lewis, R.J.; Christie, M.J. High-voltage-activated calcium current subtypes in mouse DRG neurons adapt in a subpopulation-specific manner after nerve injury. *J. Neurophysiol.* **2015**, *113*, 1511–1519. https://doi.org/10.1152/jn.00608.2014.
30. Butt, A.M.; Vanzulli, I.; Papanikolaou, M.; De La Rocha, I.C.; Hawkins, V.E. Metabotropic glutamate receptors protect oligodendrocytes from acute ischemia in mouse optic nerve. *Neurochem. Res.* **2017**, *42*, 2468–2478. https://doi.org/10.1007/s11064-017-2220-1.
31. Simmonds, M.A. Depolarizing responses to glycine, beta-alanine and muscimol in isolated optic nerve and cuneate nucleus. *Br. J. Pharmacol.* **1983**, *79*, 799–806. https://doi.org/10.1111/j.1476-5381.1983.tb10018.x.
32. Pittock, S.J.; Lucchinetti, C.F.; Parisi, J.E.; Benarroch, E.E.; Mokri, B.; Stephan, C.L.; Kim, K.; Kilimann, M.W.; Lennon, V.A. Amphiphysin autoimmunity: Paraneoplastic accompaniments. *Ann. Neurol.* **2005**, *58*, 96–107. https://doi.org/10.1002/ana.20529.

171 antigens identified initial screening, 25 antigens previously associated with glaucoma, and 24 antigens expressed in the optic nerve and astrocytes (total 220 antigens) were loaded onto custom-designed WPA plates.

Supporting Table 2. Antibody titers and positivity rates by disease type for 99 autoantibodies that could be detected by custom arrays

Anti body_gene symbol	cat AU	OAG AU	NTG AU	POAG AU	p(t-test) cat vs OAG	p(t-test) NTG vs POAG	p(t-test) NTG vs cat	p(t-test) POAG vs cat	cat PR(%)	OAG PR(%)	NTG PR(%)	POAG PR(%)
CPLX2	8.09 ± 9.69	18.46 ± 25.00	18.37 ± 28.09	18.52 ± 22.72	0.000	0.975	0.609	0.932	28.571	50.42	50.00	50.72
NEXN	4.58 ± 11.37	10.52 ± 18.71	12.33 ± 24.64	9.22 ± 12.87	0.023	0.418	0.020	0.001	8.571	30.25	28.00	31.88
MAP7D2	5.16 ± 9.07	9.23 ± 14.99	6.74 ± 11.10	11.03 ± 17.12	0.051	0.100	0.551	0.004	14.286	24.37	16.00	30.43
GATAD1	8.08 ± 19.29	9.28 ± 19.35	10.48 ± 23.72	8.40 ± 15.56	0.748	0.589	0.409	0.625	17.143	20.17	22.00	18.84
SUN1	1.56 ± 4.28	6.67 ± 18.34	2.43 ± 9.03	9.74 ± 22.40	0.006	0.016	0.296	0.165	5.714	15.13	8.00	20.29
VMAC	0.80 ± 2.06	6.34 ± 12.26	2.73 ± 6.65	8.95 ± 14.58	0.000	0.002	0.055	0.064	0.000	12.61	4.00	18.84
SOX2	4.04 ± 2.13	6.94 ± 15.26	5.25 ± 13.38	8.17 ± 16.47	0.047	0.290	0.798	0.596	0.000	12.61	6.00	17.39
CD320	7.67 ± 25.33	5.22 ± 17.90	2.67 ± 8.34	7.07 ± 22.30	0.597	0.137	0.331	0.445	11.429	12.61	10.00	14.49
CPLX1	1.46 ± 3.82	3.95 ± 7.27	3.57 ± 7.48	4.23 ± 7.16	0.008	0.629	0.474	0.024	2.857	11.76	12.00	11.59
IL1A	6.01 ± 12.99	3.21 ± 7.29	2.24 ± 4.33	3.91 ± 8.80	0.229	0.176	0.435	0.986	20.000	10.92	8.00	13.04
FLJ46010AAAF	5.06 ± 9.50	3.80 ± 9.21	3.71 ± 9.35	3.87 ± 9.17	0.490	0.926	0.120	0.108	14.286	10.08	8.00	11.59
RCOR1	2.86 ± 5.20	3.07 ± 5.80	2.35 ± 4.14	3.60 ± 6.74	0.835	0.216	0.551	0.912	11.429	10.08	8.00	11.59
MFAP1	0.55 ± 1.62	2.94 ± 5.67	2.51 ± 5.12	3.25 ± 6.06	0.000	0.478	0.355	0.918	0.000	9.24	10.00	8.70
AGAP3	2.09 ± 3.65	4.95 ± 16.20	3.31 ± 8.84	6.14 ± 19.89	0.077	0.297	0.268	0.907	2.857	9.24	8.00	10.14
NAP1L3	1.48 ± 2.48	3.37 ± 8.35	2.10 ± 4.58	4.30 ± 10.19	0.032	0.116	0.245	0.428	0.000	8.40	4.00	11.59
ZNF330	2.30 ± 9.85	4.40 ± 13.59	4.04 ± 10.32	4.67 ± 15.61	0.315	0.790	0.152	0.920	5.714	8.40	12.00	5.80
USHBP1	3.70 ± 7.31	4.30 ± 10.12	4.65 ± 13.19	4.04 ± 7.22	0.702	0.768	0.673	0.824	14.286	8.40	10.00	7.25
AKAP4	3.30 ± 11.38	1.52 ± 7.02	1.83 ± 8.26	1.29 ± 6.02	0.384	0.696	0.804	0.594	8.571	7.56	10.00	5.80
GPRC5A	5.84 ± 10.02	5.24 ± 18.91	3.94 ± 7.94	6.18 ± 23.94	0.806	0.471	0.425	0.033	20.000	7.56	8.00	7.25
ETNK1	0.83 ± 1.05	3.93 ± 16.79	1.40 ± 2.06	5.77 ± 21.86	0.047	0.104	0.514	0.385	0.000	6.72	2.00	10.14
ZRSR2	-3.42 ± 3.69	-1.71 ± 12.68	-1.43 ± 15.54	-1.91 ± 10.24	0.196	0.848	0.128	0.424	2.857	6.72	6.00	7.25
PGAM5	2.07 ± 3.62	2.71 ± 4.36	1.75 ± 3.48	3.41 ± 4.81	0.384	0.032	0.406	0.053	8.571	6.72	4.00	8.70
CEBPA	2.63 ± 5.21	3.02 ± 6.88	2.35 ± 5.53	3.50 ± 7.72	0.723	0.345	0.346	0.435	5.714	6.72	6.00	7.25
ABCF2	0.13 ± 1.64	1.61 ± 4.22	0.53 ± 2.91	2.39 ± 4.83	0.002	0.010	0.385	0.105	0.000	5.88	6.00	5.80
SLFNL1	1.22 ± 2.27	1.92 ± 4.00	1.28 ± 4.39	2.39 ± 3.65	0.191	0.147	0.517	0.542	0.000	5.88	4.00	7.25
LCOR	1.38 ± 2.17	2.70 ± 4.89	2.37 ± 5.51	2.94 ± 4.42	0.024	0.547	0.316	0.352	0.000	5.04	4.00	5.80
GATA6	1.66 ± 1.82	2.58 ± 4.78	2.42 ± 6.10	2.69 ± 3.57	0.087	0.781	0.169	0.550	0.000	5.04	6.00	4.35
PRRT2	3.48 ± 9.85	1.88 ± 6.42	2.58 ± 9.24	1.37 ± 3.05	0.369	0.375	0.017	0.086	11.429	5.04	6.00	4.35
PITX2	3.11 ± 7.31	3.34 ± 4.73	1.67 ± 1.93	4.54 ± 5.71	0.862	0.000	0.106	0.393	2.857	5.04	0.00	8.70
LIPE	0.30 ± 1.57	1.90 ± 7.67	1.63 ± 5.64	2.09 ± 8.89	0.035	0.728	0.097	0.065	0.000	4.20	6.00	2.90
HMBOX1	0.46 ± 3.10	1.04 ± 3.78	0.98 ± 4.84	1.09 ± 2.81	0.356	0.888	0.040	0.347	2.857	4.20	6.00	2.90
VPS72	2.21 ± 3.01	2.75 ± 9.52	2.90 ± 11.02	2.63 ± 8.34	0.593	0.885	0.672	0.705	2.857	4.20	4.00	4.35
ZBTB12	0.24 ± 0.84	1.58 ± 6.40	1.00 ± 3.69	2.00 ± 7.80	0.028	0.354	0.228	0.330	0.000	3.36	4.00	2.90
ZSCAN16	0.62 ± 1.08	1.09 ± 2.68	1.22 ± 3.32	1.00 ± 2.12	0.124	0.674	0.059	0.000	0.000	3.36	8.00	0.00
ZC3H8	0.45 ± 1.51	0.85 ± 3.18	0.54 ± 2.30	1.07 ± 3.68	0.305	0.333	0.141	0.223	0.000	3.36	4.00	2.90
MAGEB6	1.14 ± 3.32	1.64 ± 6.72	2.31 ± 9.71	1.15 ± 3.13	0.552	0.421	0.708	0.458	2.857	3.36	6.00	1.45
SHANK2	1.32 ± 4.89	1.48 ± 5.74	0.63 ± 2.01	2.10 ± 7.30	0.875	0.115	0.070	0.447	2.857	3.36	2.00	4.35
SKI	1.71 ± 4.56	1.77 ± 4.24	0.75 ± 1.93	2.52 ± 5.22	0.944	0.011	0.913	0.273	2.857	3.36	0.00	5.80
GPRIN1	0.56 ± 0.94	1.38 ± 3.85	1.68 ± 5.60	1.16 ± 1.76	0.037	0.522	0.533	0.044	0.000	2.52	6.00	0.00
NAT16	0.64 ± 0.93	2.07 ± 8.69	3.37 ± 13.19	1.12 ± 1.86	0.081	0.237	0.151	0.079	0.000	2.52	4.00	1.45
CHAF1B	1.09 ± 1.24	2.38 ± 7.99	3.05 ± 11.67	1.90 ± 3.49	0.091	0.502	0.633	0.538	0.000	2.52	2.00	2.90
EXD2	0.32 ± 1.38	1.91 ± 10.45	3.00 ± 15.82	1.12 ± 2.76	0.109	0.410	0.239	0.051	0.000	2.52	4.00	1.45
MNT	2.79 ± 7.07	1.03 ± 3.91	0.71 ± 5.08	1.26 ± 2.79	0.165	0.496	0.941	0.099	8.571	2.52	2.00	2.90
KLHL7	-0.21 ± 0.83	3.88 ± 32.49	-0.40 ± 0.81	6.97 ± 42.53	0.173	0.155	0.386	0.277	0.000	2.52	0.00	4.35
PCGF6	0.11 ± 0.99	0.55 ± 3.73	0.82 ± 5.19	0.35 ± 2.14	0.249	0.545	0.037	0.920	0.000	2.52	4.00	1.45
PRR20A	0.97 ± 2.09	1.80 ± 6.88	1.26 ± 4.01	2.19 ± 8.38	0.251	0.422	0.234	0.230	0.000	2.52	4.00	1.45
CTRB1	0.21 ± 1.02	0.79 ± 5.22	-0.16 ± 0.89	1.47 ± 6.75	0.260	0.050	0.037	0.108	0.000	2.52	0.00	4.35
EID3	3.17 ± 8.50	1.48 ± 7.57	0.60 ± 2.26	2.12 ± 9.73	0.294	0.216	0.247	0.098	8.571	2.52	2.00	2.90
SF3B1	1.27 ± 1.70	1.78 ± 4.41	1.33 ± 5.49	2.10 ± 3.42	0.305	0.381	0.435	0.347	0.000	2.52	2.00	2.90
ANKLE2	-4.23 ± 2.13	-3.83 ± 5.26	-4.33 ± 5.65	-3.46 ± 4.96	0.501	0.387	0.611	0.066	0.000	2.52	2.00	2.90
TWIST1	1.02 ± 2.54	1.39 ± 4.19	0.83 ± 3.92	1.79 ± 4.36	0.524	0.212	0.808	0.185	2.857	2.52	2.00	2.90
SHOX2	3.07 ± 8.72	2.05 ± 7.63	0.89 ± 1.50	2.88 ± 9.88	0.532	0.104	0.083	0.133	5.714	2.52	0.00	4.35
AMOT	2.80 ± 4.53	2.57 ± 3.59	1.28 ± 2.01	3.50 ± 4.16	0.782	0.000	0.245	0.422	5.714	2.52	2.00	2.90
MAGEB3	-0.66 ± 5.36	-0.51 ± 6.54	-1.98 ± 1.51	0.56 ± 8.36	0.888	0.016	0.322	0.319	2.857	2.52	0.00	4.35
LIMA1	0.47 ± 1.25	1.53 ± 6.52	0.32 ± 1.46	2.40 ± 8.39	0.099	0.048	0.647	0.000	0.000	1.68	0.00	2.90
CCDC136	2.33 ± 6.07	1.02 ± 2.47	0.51 ± 1.95	1.40 ± 2.75	0.220	0.041	0.117	0.143	8.571	1.68	2.00	1.45
TCEANC	0.64 ± 0.68	1.18 ± 4.64	1.39 ± 5.38	1.02 ± 4.05	0.222	0.683	0.580	0.634	0.000	1.68	2.00	1.45
C19orf47	0.29 ± 1.12	0.76 ± 3.99	0.09 ± 1.49	1.24 ± 5.05	0.252	0.078	0.263	0.314	0.000	1.68	0.00	2.90
TRIM21(1-286)	1.64 ± 5.18	6.09 ± 45.43	4.04 ± 25.01	7.57 ± 55.91	0.298	0.643	0.351	0.698	5.714	1.68	2.00	1.45
KLHL12	5.98 ± 33.52	0.45 ± 1.62	0.21 ± 0.72	0.62 ± 2.03	0.336	0.127	0.811	0.499	2.857	1.68	0.00	2.90
BANK1	8.74 ± 37.73	2.47 ± 12.27	1.19 ± 1.76	3.41 ± 16.02	0.340	0.258	0.089	0.571	5.714	1.68	0.00	2.90
CCDC102B	0.69 ± 2.01	1.11 ± 3.69	0.21 ± 1.55	1.77 ± 4.57	0.374	0.010	0.026	0.890	2.857	1.68	0.00	2.90
NCAPH2	0.37 ± 1.32	0.69 ± 5.51	-0.02 ± 1.72	1.21 ± 7.07	0.565	0.166	0.024	0.607	0.000	1.68	2.00	1.45
TRIM21(1-400)	2.96 ± 8.49	4.81 ± 32.23	3.89 ± 23.46	5.48 ± 37.48	0.574	0.777	0.516	0.333	8.571	1.68	2.00	1.45
CLCN5	0.85 ± 2.29	1.25 ± 6.76	2.09 ± 10.21	0.63 ± 1.79	0.589	0.322	0.547	0.316	0.000	1.68	4.00	0.00
ZSCAN18	0.39 ± 3.25	0.74 ± 5.81	0.15 ± 2.37	1.17 ± 7.35	0.650	0.285	0.093	0.011	2.857	1.68	2.00	1.45
PEX5L	1.62 ± 5.61	1.18 ± 3.62	1.36 ± 3.08	1.05 ± 3.98	0.664	0.633	0.015	0.822	5.714	1.68	2.00	1.45
MAGEB18	0.30 ± 0.68	1.01 ± 3.32	0.75 ± 1.62	1.20 ± 4.14	0.030	0.410	0.908	0.130	0.000	0.84	0.00	1.45
MRPL1	0.32 ± 0.86	0.84 ± 2.85	0.61 ± 0.76	1.01 ± 3.69	0.083	0.381	0.687	0.117	0.000	0.84	0.00	1.45
DDX6	1.16 ± 0.82	1.79 ± 5.27	1.12 ± 0.92	2.28 ± 6.85	0.213	0.167	0.254	0.017	0.000	0.84	0.00	1.45
SSSCA1	0.12 ± 1.00	1.71 ± 17.87	3.79 ± 27.56	0.21 ± 1.29	0.334	0.362	0.164	0.369	0.000	0.84	2.00	0.00
ZNF713	0.66 ± 1.65	0.39 ± 1.55	-0.08 ± 0.61	0.74 ± 1.91	0.404	0.001	0.945	0.460	0.000	0.84	0.00	1.45
TROVE2	0.08 ± 1.10	0.75 ± 8.60	-0.17 ± 0.52	1.42 ± 11.27	0.405	0.247	0.013	0.001	0.000	0.84	0.00	1.45
WBP4	1.95 ± 8.21	0.56 ± 10.09	1.65 ± 15.39	-0.24 ± 2.15	0.406	0.393	0.672	0.223	2.857	0.84	2.00	0.00
DBT	0.22 ± 1.36	0.48 ± 3.66	-0.02 ± 0.41	0.84 ± 4.77	0.525	0.142	0.089	0.569	0.000	0.84	0.00	1.45
TRAM1	0.11 ± 2.12	0.46 ± 4.40	0.68 ± 6.68	0.30 ± 1.18	0.526	0.695	0.093	0.583	2.857	0.84	2.00	0.00
SSB	-0.19 ± 0.87	0.24 ± 7.58	-0.51 ± 0.99	0.77 ± 9.92	0.546	0.290	0.165	0.068	0.000	0.84	0.00	1.45
TROVE2	0.40 ± 1.16	0.72 ± 5.75	-0.05 ± 0.51	1.27 ± 7.51	0.567	0.247	0.663	0.255	0.000	0.84	0.00	1.45
EDA2R	0.48 ± 1.77	0.64 ± 2.12	0.85 ± 2.41	0.49 ± 1.89	0.639	0.392	0.037	0.394	0.000	0.84	2.00	0.00
CHST12	0.39 ± 1.39	0.53 ± 2.26	0.07 ± 0.76	0.87 ± 2.86	0.645	0.030	0.266	0.841	0.000	0.84	0.00	1.45
OXTR	0.68 ± 0.92	0.60 ± 2.28	0.71 ± 3.18	0.51 ± 1.31	0.754	0.680	0.416	0.001	0.000	0.84	2.00	0.00
IKZF3	0.33 ± 0.97	0.29 ± 1.68	0.18 ± 1.27	0.36 ± 1.93	0.849	0.548	0.224	0.252	0.000	0.84	0.00	1.45
MECP2	-0.43 ± 0.69	-0.08 ± 1.05	-0.50 ± 0.66	0.23 ± 1.17	0.021	0.000	0.244	0.087	0.000	0.00	0.00	0.00
ZNF253	0.77 ± 1.29	0.24 ± 1.16	0.15 ± 0.88	0.30 ± 1.33	0.033	0.478	0.413	0.958	0.000	0.00	0.00	0.00
DPYSL4	0.73 ± 2.52	-0.11 ± 1.16	-0.24 ± 1.05	-0.01 ± 1.23	0.064	0.271	0.353	0.450	2.857	0.00	0.00	0.00
TRIM38	0.66 ± 0.95	0.39 ± 0.78	0.45 ± 0.53	0.35 ± 0.92	0.130	0.437	0.500	0.137	0.000	0.00	0.00	0.00
SPTA1	-0.46 ± 1.08	-0.81 ± 1.70	-1.07 ± 1.35	-0.62 ± 1.89	0.155	0.126	0.082	0.081	0.000	0.00	0.00	0.00
ZNF710	1.58 ± 2.18	1.02 ± 1.44	0.74 ± 0.94	1.22 ± 1.70	0.157	0.052	0.429	0.523	2.857	0.00	0.00	0.00
PHF1	0.79 ± 3.03	0.23 ± 1.26	-0.12 ± 0.94	0.49 ± 1.40	0.296	0.005	0.119	0.746	2.857	0.00	0.00	0.00
HHLA3	0.47 ± 1.68	0.15 ± 1.64	-0.25 ± 1.34	0.44 ± 1.78	0.314	0.017	0.307	0.352	0.000	0.00	0.00	0.00
ATP4A	-3.43 ± 9.69	-5.02 ± 3.03	-5.81 ± 3.12	-4.44 ± 2.85	0.347	0.016	0.232	0.344	2.857	0.00	0.00	0.00
ARHGAP39	0.35 ± 1.90	0.04 ± 1.63	-0.47 ± 1.13	0.41 ± 1.84	0.380	0.002	0.605	0.786	0.000	0.00	0.00	0.00
SLTM	-0.06 ± 0.43	-0.16 ± 0.92	-0.22 ± 0.47	-0.12 ± 1.15	0.399	0.498	0.790	0.258	0.000	0.00	0.00	0.00
CRBN	1.21 ± 4.41	0.57 ± 1.42	0.50 ± 0.76	0.62 ± 1.76	0.403	0.622	0.170	0.027	2.857	0.00	0.00	0.00
IKZF1	1.21 ± 1.67	1.01 ± 1.58	0.83 ± 1.32	1.14 ± 1.74	0.530	0.272	0.830	0.226	0.000	0.00	0.00	0.00
ZSCAN31	0.20 ± 1.34	0.09 ± 1.49	-0.30 ± 1.27	0.37 ± 1.58	0.675	0.012	0.243	0.112	0.000	0.00	0.00	0.00
GTF2B	0.34 ± 0.50	0.34 ± 1.13	0.28 ± 0.45	0.39 ± 1.44	0.956	0.561	0.093	0.389	0.000	0.00	0.00	0.00
GIF	0.40 ± 0.55	0.41 ± 1.00	0.59 ± 1.16	0.27 ± 0.84	0.957	0.098	0.941	0.048	0.000	0.00	0.00	0.00

AU and PR were calculated for each disease for the 99 autoantigens detected in the custom-designed WPA.

Supporting Table 3. ROC analysis of each disease type

Cataract vs. OAG

	AUC	95% CI	Cut Off Value	Sensitivity	Specificity
ETNK1	0.728	0.639-0.817	1.350	0.886	0.563
VMAC	0.796	0.717-0.876	2.050	0.914	0.630
NEXN	0.594	0.496-0.691	5.400	0.800	0.437
SUN1	0.553	0.448-0.658	0.650	0.686	0.471

Cataract vs. NTG

	AUC	95% CI	Cut Off Value	Sensitivity	Specificity
ETNK1	0.601	0.479-0.724	0.750	0.600	0.600
VMAC	0.669	0.615-0.832	0.800	0.714	0.660
NEXN	0.551	0.428-0.673	14.000	0.943	0.260
SUN1	0.467	0.343-0.591	0.550	0.629	0.440

Cataract vs. POAG

	AUC	95% CI	Cut Off Value	Sensitivity	Specificity
ETNK1	0.820	0.733-0.907	1.350	0.886	0.754
VMAC	0.889	0.818-0.959	2.100	0.914	0.826
NEXN	0.625	0.516-0.735	5.400	0.800	0.493
SUN1	0.615	0.500-0.731	0.650	0.686	0.551

Summarization of ROC parameters for each autoantibody to discriminate the glaucoma groups from the cataract (control) group.

Supporting Table 4. Spearman rank correlation between autoantibodies in OAG

	NEXN	SUN	ETNK1	VMAC
NEXN	1	0.102	-0.024	0.177
SUN1	0.102	1	0.111	0.296
ETNK1	-0.024	0.111	1	0.283
VMAC	0.177	0.296	0.283	1

Correlation between each autoantibody was examine by Spearman rank correlation test.

Supporting Table 5. ROC logistic analysis between glaucoma and cataract Comparison of ROC

		AUC	95% CI	p-Value vs. Model 1	Cut Off Value	Sensitivity	Specificity
Cataract vs. OAG	Model 1	0.566	0.445-0.687	-	0.773	0.580	0.571
	Model 2	0.828	0.757-0.898	0.0002*	0.735	0.748	0.829
Cataract vs. NTG	Model 1	0.657	0.537-0.777	-	0.614	0.580	0.686
	Model 2	0.757	0.652-0.863	0.0637	0.521	0.82	0.71
Cataract vs. POAG	Model 1	0.630	0.520-0.740	-	0.647	0.623	0.571
	Model 2’	0.929	0.875-0.983	< 0.0001*	0.520	0.899	0.857

Summarization of parameters of logistic regression analysis to determine usefulness of a series of autoantibody combinations as diagnostic tests in discriminating OAG, NTG, and POAG from cataracts.

Supporting figure 1. Representative image of proteins bound on the CWPA

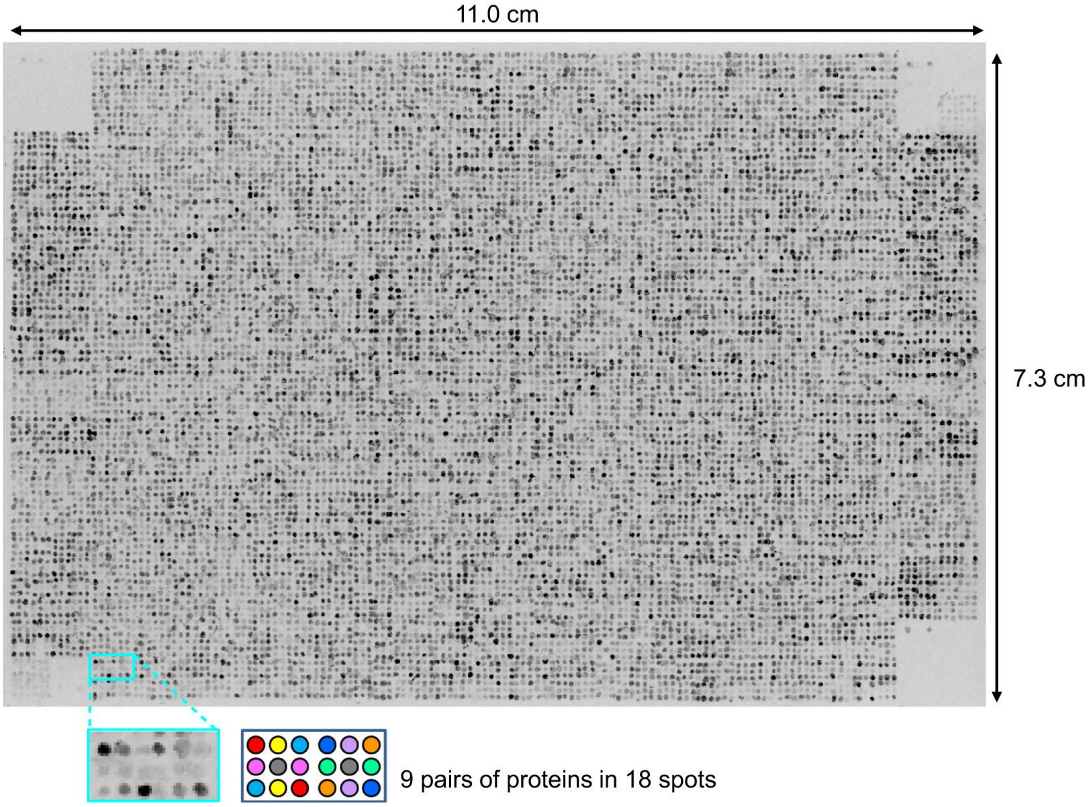

*Reference: Fukuda E, Tanaka H, Yamaguchi K, et al. Identification and characterization of the antigen recognized by the germ cell mAb TRA98 using a human comprehensive wet protein array. Genes Cells 2021;26(3):180-9.

Comprehensive Wet Protein Array (CWPA) were performed as initial screening of autoantibodies. The representative fluorescent image was represented.

Supporting Figure 2. Relationship between optimal cutoff value by Youden Index, sensitivity, and specificity

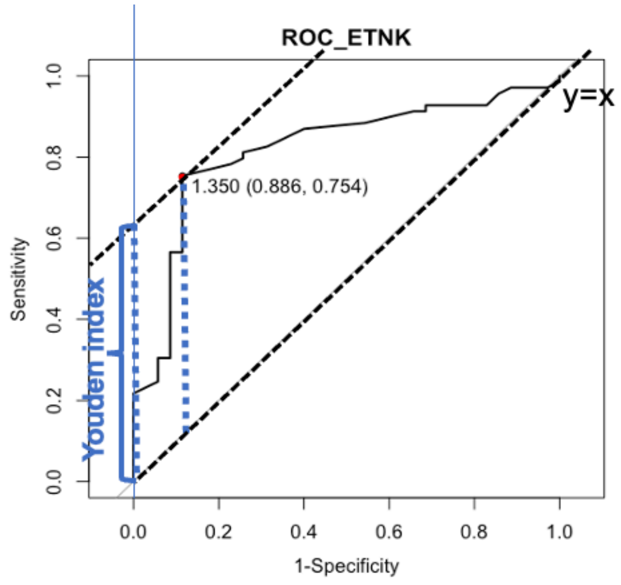

In the ROC analysis for discriminating cataract and POAG groups using ETNK1, the maximum value of "sensitivity - (1 - specificity)" (Youden Index) is the point where the distance between the line graph and the diagonal line, (length of the dotted line perpendicular to the diagonal line) is the greatest, and represents the optimal cutoff value (red point) that separates cataracts from NTG. The dark dashed line is y=x and the light dashed line is y=x + the maximal Youden Index passing through the red point (the optimal cutoff value). Arrow indicates the maximal Youden Index.

Supporting Figure 3. Autoantibody overlap in NTG (A) and POAG (B)

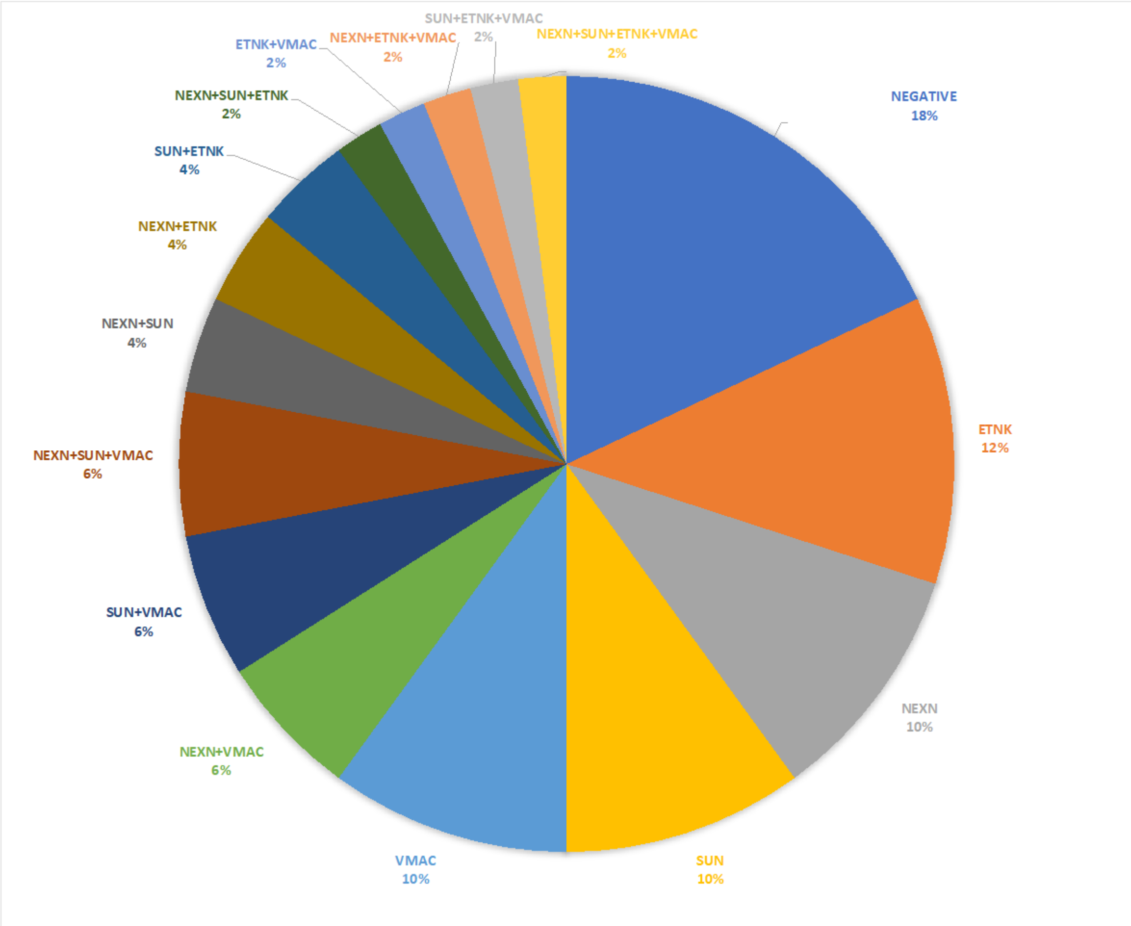

A

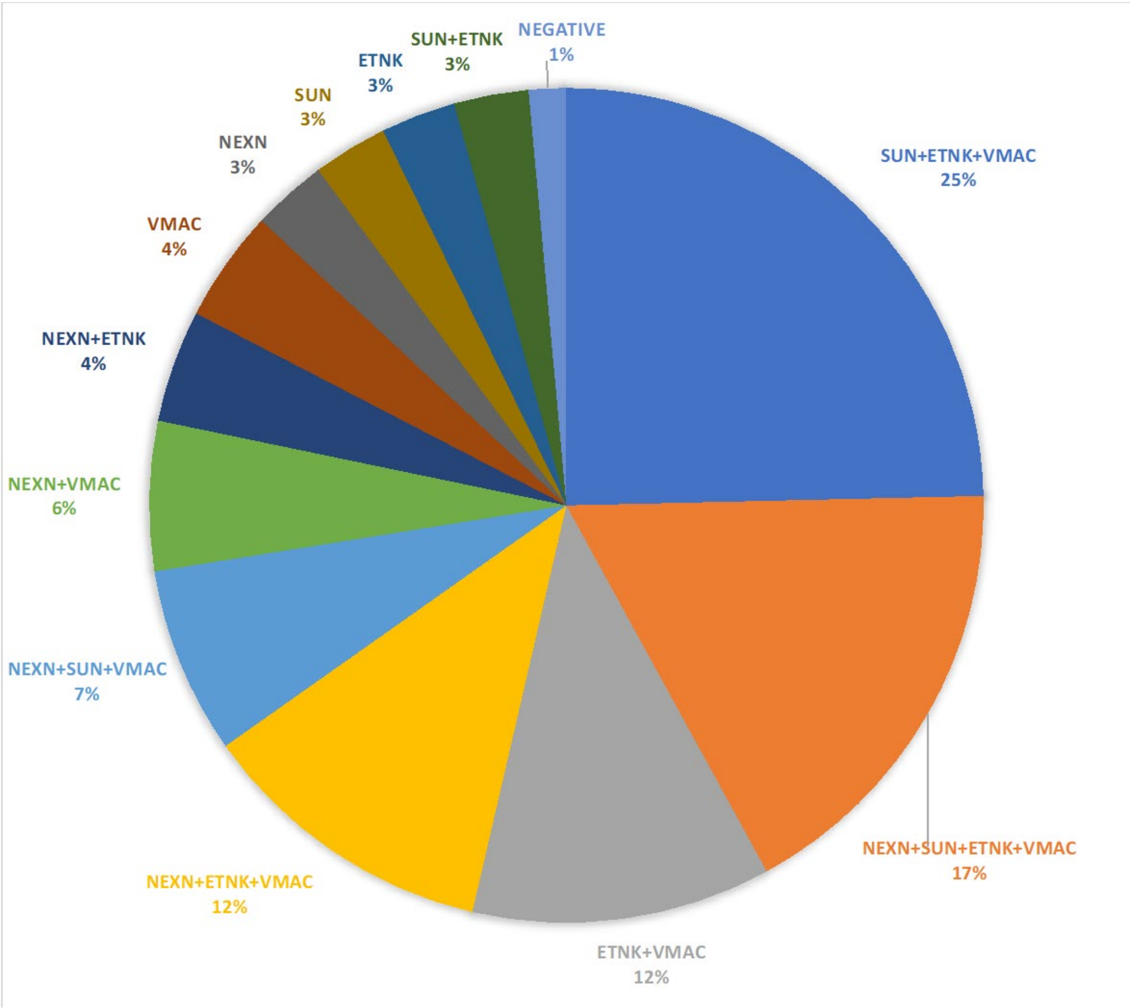

B

Overlap of autoantibody is examined in NTG and POAG.

Supporting Figure 4. Comparison of positivity rates between early stage and progress stage of glaucoma

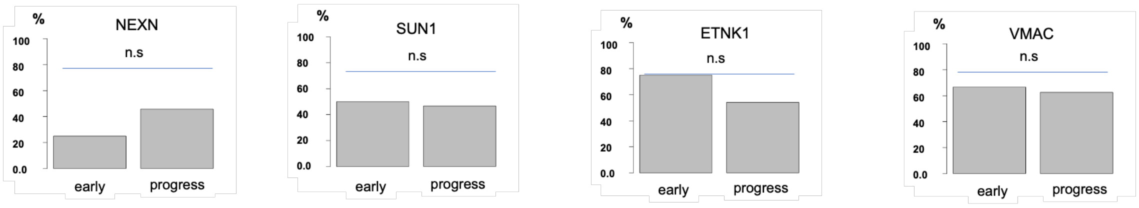

	Early (n=12)	Progress (n=107)	p value (Fisher test)
NEXN	3 (25%)	49 (45.8%)	0.23
SUN1	6 (50%)	50 (46.7%)	1
ETNK1	9 (75%)	58 (54.2%)	0.23
SOX2	7 (58.3%)	50 (46.7%)	0.55
VMAC	8 (66.7%)	67 (62.6%)	1

Positivity rates between early stage and more progressed stage glaucoma were compared to confirm the usefulness of each antibody in detection of early stage glaucoma.

Supporting Figure 5. Diagnostic criteria for glaucoma

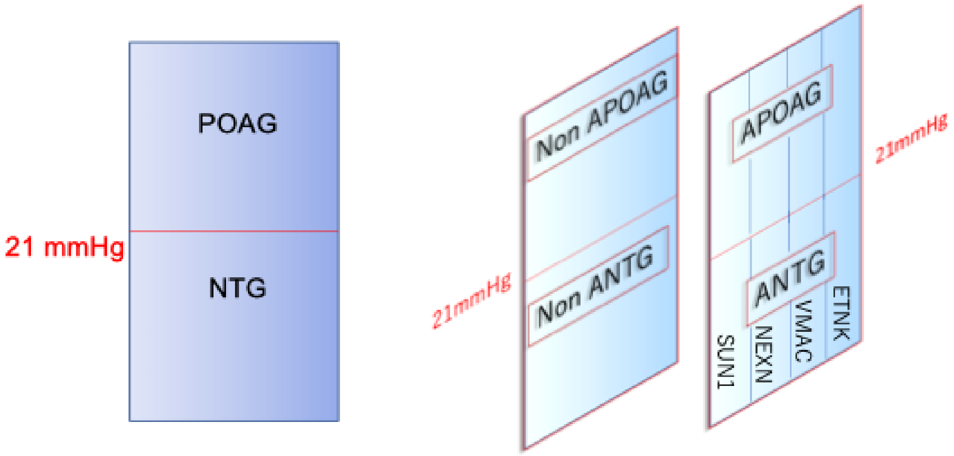

A **B**

Classification in OAG. **A.** OAG has been divided into POAG and NTG according to IOP 21 mmHg. **B.** OAG can be classified into four categories according to the presence or absence of autoantibodies: Autoantibody-related POAG (APOAG), Non-APOAG, Autoantibody-related NTG (ANTG) and Non-ANTG (B). In the future, ANTG may be further subdivided according to its antibody type.
